# BRIDGING PERSPECTIVES: A COMPARATIVE EXAMINATION OF SUBJECTIVE MEMORY AND AGING MEASURES

**DOI:** 10.1093/geroni/igae098.0025

**Published:** 2024-12-31

**Authors:** Steven Harris, Jacklyn Gehling, Hannah Marx, Ravneet Dhaliwal, Frank Robertson, Claudia Jacova Chenoweth

**Affiliations:** Pacific University, Hillsboro, Oregon, United States; Pacific University, Hillsboro, Oregon, United States; Pacific University, Hillsboro, Oregon, United States; Pacific University, Hillsboro, Oregon, United States; Pacific University, Hillsboro, Oregon, United States; Pacific University, Hillsboro, Oregon, United States

## Abstract

Understanding subjective cognition is imperative as it offers valuable insights into individuals’ perceptions, beliefs, and interpretations regarding their cognitive abilities, exerting a profound influence on their overall well-being and behavior. This study sought to explore perceptions of subjective cognition and aging among a national sample of community-dwelling older adults (n=279, M=73.8, education M=14.9, 52% female). In particular, we evaluated relationships between three measures of subjective cognition and aging: subjective memory complaints, subjective memory age, and subjective age. Everyday Cognition-Memory (ECog-mem) items were summed to measure memory complaints; memory age identity (MAI) was obtained by subtracting subjective memory age from chronological age; Subjective age identity (SAI) was calculated by subtracting subjective age from chronological age. We also assessed subjective health and objective memory using AVLT-Delayed recall. We conducted three hierarchical regression analyses to predict ECog-mem, MAI, and SAI. In each model, we entered age and education in the first step, subjective health and objective memory (AVLT) in the second, and depression (PHQ-2) in the third. Our sample reported a low burden of memory complaints (M=12.35) and a younger subjective than chronological age: MAI (M=-10.82) and SAI (M=-9.86). These measures were only moderately correlated (range r.29-.64). Better health ratings predicted lower ECog-mem (B=1.387, p<.001), younger MAI (B=4.16, p<.001), and younger SAI (B=4.72, p<.001), and these contributions remained after PHQ2 entry. Objective memory did not contribute to the prediction. Subjective cognition and aging measures appear to capture somewhat different experiences, but these are all influenced by perceptions of health.

